# Enhanced motivation of cognitive control in Parkinson's disease

**DOI:** 10.1111/ejn.14137

**Published:** 2018-09-16

**Authors:** Monique H.M. Timmer, Esther Aarts, Rianne A.J. Esselink, Roshan Cools

**Affiliations:** ^1^ Donders Institute for Brain Cognition and Behaviour Centre for Cognitive Neuroimaging Radboud University Nijmegen The Netherlands; ^2^ Department of Neurology and Parkinson Centre Nijmegen (ParC) Radboud University Medical Centre Nijmegen The Netherlands; ^3^ Department of Psychiatry Radboud University Medical Centre Nijmegen The Netherlands

**Keywords:** depression, dopamine, reward, task‐switching

## Abstract

Motor and cognitive deficits in Parkinson's disease (PD) have been argued to reflect motivational deficits. In prior work, however, we have shown that motivation of cognitive control is paradoxically potentiated rather than impaired in Parkinson's disease. This is particularly surprising given the fact that Parkinson's disease is often accompanied by depression, a prototypical disorder of motivation. To replicate our previous finding and assess the effects of depression, we investigated performance of PD patients with (*n* = 22) and without depression (history) (*n* = 23) and age‐matched healthy controls (*n* = 23) on a task specifically designed to measure the effect of reward motivation on task‐switching. We replicated previous findings by showing contrasting effects of reward motivation on task‐switching in PD patients and age‐matched healthy controls. While the promise of high versus low reward improved task‐switching in PD, it tended to impair task‐switching in age‐matched healthy controls. There were no effects of a depression (history) diagnosis in PD patients. These findings reinforce prior observations that Parkinson's disease is accompanied by enhanced incentive motivation of cognitive control and highlight the potential of incentive motivational strategies for overcoming cognitive deficits in Parkinson's disease.

**Abbreviations**
AS: Apathy Scale
BDI: Beck Depression Inventory
FMT‐PET: 6‐[(18)F]‐fluoro‐L‐m‐tyrosine Positron Emission Tomography
LED: Levodopa Equivalent Dose
MMSE: Mini Mental State Examination
NART: National Adult Reading Test
PD: Parkinson's Disease
SPECT: Single Photon Emission Computed Tomography
STAI: Spielburg State Trait Anxiety Inventory
UPDRS‐III: Unified Parkinson's Disease Rating Scale, motor part

## 1 INTRODUCTION

Parkinson's disease (PD) is a progressive neurodegenerative disorder, characterized by severe dopamine depletion in the striatum, as well as abnormalities in other neuromodulatory systems. It is accompanied primarily by motor symptoms, such as bradykinesia and motor rigidity, but many patients also exhibit significant dopamine‐dependent cognitive deficits. Dopamine‐dependent cognitive deficits, for example, in task‐switching and working memory, are seen even in the early stages of the disease, and in a manner that is independent from dementia (Owen et al., 1995; Kehagia, Barker, & Robbins, 2010; Robbins & Cools, 2014).

Consistent with an original characterization of PD as a paralysis of the will, the motor deficits, in particular bradykinesia, have been argued to reflect a motivational deficit (Niv & Rivlin‐Etzion, 2007). For example, Mazzoni and colleagues demonstrated that, although PD patients can display motor behaviour that matches that of healthy controls in both speed and accuracy, they performed motor actions with lower probability (Mazzoni, Hristova, & Krakauer, 2007). This finding indicated that the movement slowing characteristic of PD reflects a motivational or cost–benefit decision deficit rather than a pure motor deficit (Niv, Daw, Joel, & Dayan, 2007). This concurred with subsequent optimal control theory‐based work, suggesting that the main determinant of the movement deficit in PD is reduced optimization of motor effort (Baraduc, Thobois, Gan, Broussolle, & Desmurget, 2013). This motor motivation hypothesis, which implies that PD patients do not “want” to move, rather than not being able to move, was further strengthened by a series of recent studies with PD patients, showing reduced reward sensitivity of both speed and accuracy of motor decisions (Manohar et al., 2015, but see Kojovic et al., 2014), as well as decreased willingness to exert motor effort (Porat, Hassin‐Baer, Cohen, Markus, & Tomer, 2014; Chong et al., 2015; Le Bouc et al., 2016). In keeping with the well‐known implication of dopamine in motivation and cost–benefit decision making (Berridge, 2007; Cools, 2008; Collins & Frank, 2014; Salamone, Yohn, Lopez‐Cruz, Miguel, & Correa, 2016), the latter studies confirmed that the willingness to exert motor effort depends on dopaminergic medication status, with patients exhibiting reduced motor motivation in the OFF medication state compared with the ON medication state (Porat et al., 2014; Chong et al., 2015; Le Bouc et al., 2016).

An open question is whether PD is also accompanied by reduced motivation of cognitive control. Based on the above‐reviewed literature on motor motivation in PD, one might expect that PD patients also exhibit reduced reward sensitivity of performance on cognitive control tasks. However, there is little evidence for reduced cognitive motivation and, if anything, the reverse pattern is observed. While Harsay, Buitenweg, Wijnen, Guerreiro, & Ridderinkhof (2010) reported no effects of PD on the reward sensitivity of performance on an antisaccade task, Aarts and colleagues (Aarts et al., 2011) in fact showed that relative to age‐ and IQ‐matched controls, PD patients, who were tested OFF their normal dopaminergic medication, exhibited significantly enhanced reward sensitivity of task‐switching. Critically, this effect covaried with dopamine cell loss, as indexed by (123)I‐FP‐CIT binding in the striatum measured with SPECT (Single Photon Emission Computed Tomography) (Aarts et al., 2011): Dopamine cell loss in the dorsal striatum (i.e. posterior putamen) correlated positively with the degree to which the promise of a monetary reward reduced task‐switching costs, so that PD patients with the greatest striatal dopamine depletion exhibited the greatest beneficial effect of reward on task‐switching.

This finding is not only paradoxical in light of the above‐mentioned theories that consider PD to be a disorder of the will (Mazzoni et al., 2007; Niv & Rivlin‐Etzion, 2007; Chong et al., 2015) but also in light of several other observations in PD. First, impaired cognitive control (i.e. task‐switching deficit) is a core feature of PD (Downes et al., 1989; Cools, Barker, Sahakian, & Robbins, 2001a,b, 2003; Pollux, 2004; Witt et al., 2006). Second, many PD patients suffer from non‐motor symptoms that are associated with motivational deficits, such as depression and apathy (Reijnders, Ehrt, Weber, Aarsland, & Leentjens, 2008; den Brok et al., 2015), and depression (non‐PD) has been shown to be associated with decreased reward motivation during effort‐based decision making (Treadway, Buckholtz, Schwartzman, Lambert, & Zald, 2009; Yang et al., 2014) and diminished behavioural as well as neural (striatal) responses to incentive cues (Henriques & Davidson, 2000; Knutson, Bhanji, Cooney, Atlas, & Gotlib, 2008; Stoy et al., 2012; Yang et al., 2016). And lastly, ample evidence implicates striatal dopamine in reward motivation, which is severely depleted in PD (Berridge, 2007; Aarts et al., 2010; Salamone & Correa, 2012; Salamone et al., 2016).

However, the finding does concur remarkably well with another recent finding showing a similar negative correlation between an index of striatal dopamine, dopamine synthesis capacity as measured with 6‐[(18)F]‐fluoro‐L‐m‐tyrosine Positron Emission Tomography (FMT‐PET) and motivated cognitive control in young healthy volunteers (Aarts, Wallace, et al., 2014). In these healthy controls, higher striatal dopamine in dorsal striatum (i.e. left caudate nucleus) was associated with greater detrimental effects of reward motivation on cognitive control, this time measured in terms of Stroop interference control. This finding in healthy volunteers casts our earlier observation in PD patients in a new light. The PD work showed that reward motivation potentiates cognitive control in people with severely depleted levels of dopamine in the dorsal striatum, which we interpreted at the time as (over)compensation in the relatively intact ventral striatal dopamine neurons (Aarts et al., 2011). However, the more recent finding of reward motivation undermining cognitive control in high‐dopamine controls (Aarts, Wallace, et al., 2014) rather seems to suggest a linearly negative relationship between dopamine and motivated cognition, with Parkinson's disease patients on the left side of the curve and high‐dopamine controls on the right.

Given the renewed relevance of this observation in PD patients, also in light of recent renewed interests in motivational and value‐based accounts of control (Kurzban, Duckworth, Kable, & Myers, 2013; Shenhav, Botvinick, & Cohen, 2013; Cools, 2016; Westbrook & Braver, 2016), we aimed here, first, to replicate our finding that effects of reward motivation on task‐switching are potentiated in a novel sample of non‐depressed PD patients. Moreover, in line with the proposed key role for dopamine, we also assessed whether this effect depends on dopaminergic medication state by comparing performance of patients when they were in their ON and OFF states. Furthermore, we aimed to address whether reward motivational enhancement of task‐switching in PD is abolished in PD patients with depression (history), following prior work showing reduced reward sensitivity in depression (Knutson et al., 2008; Eshel & Roiser, 2010; Roiser, Elliott, & Sahakian, 2012; Yang et al., 2014), or whether this increased motivated cognition is intact, given equally or even more diminished dopamine levels in the striatum of PD patients with depression (Remy, Doder, Lees, Turjanski, & Brooks, 2005; Weintraub et al., 2005; Rektorova, Srovnalova, Kubikova, & Prasek, 2008; Joutsa, Rinne, Eskola, & Kaasinen, 2013; Vriend et al., 2014, but see Felicio et al., 2010; Ceravolo et al., 2013). To this end, we assessed PD patients with and without depression (history) and healthy controls using a task similar to that used in previous studies (Aarts et al., 2011; Aarts, Nusselein, et al., 2014). All patients were tested twice, ON and OFF dopaminergic medication.

## 2 MATERIALS AND METHODS

### 2.1 Participants

Twenty‐four PD patients with depression (history), 23 non‐depressed PD patients and 25 healthy controls were recruited. Data from two healthy controls were discarded from the analysis because of a lifetime depression history. Furthermore, two PD patients with depression (history) failed to complete the study, leading to incomplete datasets. Reported analyses include 22 PD patients with and 23 PD patients without depression (history) and 23 healthy controls. Based on a power calculation for which we used GPower software (Cunningham & McCrum‐Gardner, 2007), a minimum of 18 participants per group was estimated sufficient to show a significant effect (with power of 0.80, α error probability of 0.05 and a medium effect size (*f* = 0.25)).

Patients were recruited from the Parkinson Center at the Radboud University Medical Centre, Nijmegen, Netherlands. Healthy controls were recruited via advertisement or were partners or acquaintances of participating patients. The three groups were matched for age, gender and IQ measured with the NART (Dutch version of the National Adult Reading Test, (Schmand, Bakker, Saan, & Louman, 1991)) (Table 1). Patient groups were also matched for disease severity measured with the UPDRS‐III (Goetz & Stebbins, 2004), disease duration (years) and amounts of dopaminergic medication (levodopa equivalent dose, (Esselink et al., 2004)) (Table 1). The study was approved by the local ethics committee (CMO regio Arnhem—Nijmegen, The Netherlands, nr. 2012/43) and written informed consent according to the Declaration of Helsinki was obtained from all participants. Participants were paid for participation according to the institutional guidelines.

**Table 1:** Patient and control group characteristics

	Non‐depressed PD (*n* = 23)	PD with depression (history) (*n* = 22)	Healthy controls (*n* = 23)
Gender, men	14	14	14
Age, years	61.0 (±7.4)	58.4 (±5.7)	60.9 (±5.9)
NART‐IQ	97.0 (±15.1)	95.7 (±11.5)	100.7 (±13.7)
Handedness, Right	18	18	20
Response hand, Right	14	7	12
MMSE	28.5 (±1.3)	28.4 (±1.4)	28.8 (±1.2)
BDI	4.1 (±2.3)	9.6 (±6.1)^a^	3.1 (±2.1)
AS	9.0 (±4.4)	13.6 (±3.9)^a^	8.6 (±2.9)
STAI	28.4 (±4.5)	37.0 (±7.0)^a^	26.8 (±3.6)
UPDRS‐III	21.8 (±6.7)	23.1 (±9.6)	
Disease duration, years	4.5 (±2.2)	5.0 (±3.5)	
LED	618.3 (±272.8)	547.7 (±242.4)	
First session ON	13	11	

Notes

PD: Parkinson's disease; NART: National Adult Reading Test (Schmand *et al*., 1991); MMSE: Mini Mental State Examination (Folstein *et al*., 1975); BDI: Beck Depression Inventory (Beck, Erbaugh, Ward, Mock, & Mendelsohn, 1961); AS: Apathy Scale (Starkstein *et al*., 1992); STAI: Spielberg State Trait Anxiety Inventory (Hedberg, 1972); UPDRS‐III: Unified Parkinson's Disease Rating Scale, motor part (Goetz *et al*., 2008); LED: levodopa equivalent dose. LED was calculated, pooling different drugs according to the following formula: regular levodopa × 1 + slow release levodopa × 0.7 + ropinirole × 20 + pramipexole × 100 + [regular levodopa dose + (slow release levodopa × 0.7)] × 0.2 if taking entacapone (Esselink *et al*., 2004).

a PD patients with depression (history) differed significantly from both non‐depressed and healthy controls (*p *< 0.001).

All patients were diagnosed with idiopathic Parkinson's disease according to the UK Brain Bank criteria (Gibb & Lees, 1988). Diagnosis were made by a neurologist specialized in Movement Disorders (Prof. B.R. Bloem, Dr. R.A. Esselink or Dr. B. Post). All patients were treated with dopaminergic medication: levodopa (depressed PD group *n* = 15, non‐depressed PD group *n* = 11), dopamine receptor agonists (depressed PD group *n* = 2, non‐depressed PD group *n* = 2) or a combination of both (depressed PD group *n* = 5, non‐depressed PD group *n* = 10). Eight patients in the depressed PD group received antidepressants (paroxetine *n* = 3, escitalopram *n* = 1, venlafaxine *n* = 1, nortriptyline *n* = 2 and citalopram *n* = 1). Patients were selected for the depressed PD group if they met the DSM‐IV criteria for a major (*n* = 7) or minor depressive episode (*n* = 13), dysthymic disorder (*n* = 1) or adjustment disorder with depressed mood (*n* = 1) within the 5 years before PD diagnosis or during their disease course up until now. Seven patients were diagnosed with current depression, the other patients with past depression. PD patients with a past depression were included in the depressed group because impaired reward motivation (and underlying striatal dysfunction) has also been shown in individuals at risk of depression, putatively reflecting an underlying vulnerability trait (Olino et al., 2014). Psychiatric diagnosis was established via structured psychiatric interviews (MINI‐plus, (Sheehan et al., 1998)) conducted during an intake session. The timeframe of 5 years was chosen because the incidence of depression is higher within the 5 years before PD diagnosis and therefore presumably related to PD pathology (Shiba et al., 2000).

General exclusion criteria were any other neurological or psychiatric disorders (bipolar disorder, schizophrenia, ADHD, drugs and/or alcohol abuse) and clinical dementia assessed with a Mini Mental State Examination (cut off of <24, (Folstein, Folstein, & McHugh, 1975)). Healthy controls were also excluded if they had a history of a mood or anxiety disorder or if they used any psychotropic medication.

### 2.2 General procedures

This experiment was conducted as part of a larger project investigating the neurobiological mechanisms of depression in Parkinson's disease. Patients and healthy controls were first scheduled for an intake session to obtain informed consent and check for inclusion and exclusion criteria. Measurements in healthy controls were obtained once, whereas measurements in PD patients were obtained twice: once while using their regular dopaminergic medication (ON) and once after withdrawal of their dopaminergic medication for at least 18 hr (24 hr for slow‐release dopamine receptor agonists) (practically defined OFF). The order of ON and OFF sessions was randomized such that approximately half of the patients, in both patient groups, were first tested ON medication (depressed PD group *n* = 11, non‐depressed PD group *n* = 13) and the other half first tested OFF medication (depressed PD group *n* = 11, non‐depressed PD group *n* = 10). Testing days were on average 22 days apart (*SD* 27.0) in the depressed PD group and 21 days (*SD* 19.8) in the non‐depressed PD group. Patients were on stable medication regimes during the course of the study, except for one patient in the depressed PD group who was shortly treated for pain (4 weeks) with duloxetine in between the two testing days. This medication was discontinued 4 weeks before the second testing day. Patients who received antidepressants were asked specifically to take this medication on both testing days to assure that within‐subject differences between testing days are attributed to dopaminergic manipulation only. All testing days started in the morning between 8:30 and 10:30 a.m.

### 2.3 Task

Participants performed a well‐established pre‐cued task‐switching paradigm designed to measure effects of reward motivation on task‐switching identical to one employed previously (Aarts et al., 2011). Participants were presented a series (240 in total) of incongruent Stroop‐like arrow‐word targets (either the word “left” in a right pointing arrow or the word “right” in a left pointing arrow). On each trial they were asked to respond either to the direction of the arrow or to the direction of the word by pressing a left or right button. Patients responded with their least affected hand, which was not always the dominant hand. Therefore, we asked some healthy controls (randomly) to respond with their non‐dominant hand as well. This was matched between groups (Table 1). Half of the trials were repeat trials and half of the trials were switch trials (switch from arrow to word target or vice versa). Furthermore, half of the trials—repeat and switch—were preceded by a high‐reward (10 cents) cue and the other half by a low‐reward (1 cent) cue, indicating the amount of money participants could obtain by responding correctly and in time. The order of trials was pseudo‐randomized. All participants were familiarized with the task directly preceding the experiment and performed two practice blocks. Response deadlines – separately for arrow, word, repeat and switch trials – were individually determined based on performance on 24 trials performed directly after practice and before the start of the experiment. Patients performed these practice blocks on both testing days and response deadlines were adjusted based on performance on that specific day.

### 2.4 Analyses

Reaction times (RTs) and error rates (proportion of errors per trial type) were analysed. For statistical purposes, RTs were log transformed to maximize homogeneity of variance between groups. An arcsine transformation (2 arcsin√x) was applied to error rates (Sheskin 2003). Untransformed data are shown in Table 2 as a function of group and medication session.

**Table 2:** Raw (untransformed) data on the rewarded task‐switching paradigm

	OFF			ON		
	Low reward	High reward	Reward benefit	Low reward	High reward	Reward benefit
Error rates (%)						
Non‐depressed PD						
Repeat	11.0 (2.4)	10.7 (2.4)	0.004 (0.012)	5.9 (1.0)	6.8 (1.1)	−0.009 (0.010)
Switch	13.3 (2.2)	11.1 (2.0)	0.022 (0.011)	10.0 (1.5)	7.1 (1.1)	0.029 (0.010)
PD with depression (history)						
Repeat	5.4 (0.9)	5.6 (1.0)	−0.002 (0.009)	5.7 (1.3)	5.8 (0.9)	−0.000 (0.008)
Switch	8.8 (1.2)	8.4 (1.1)	0.004 (0.011)	9.1 (1.4)	7.9 (1.4)	0.012 (0.013)
Healthy controls						
Repeat	7.2 (1.8)	6.3 (1.6)	0.009 (0.008)			
Switch	8.4 (1.5)	10.2 (2.0)	−0.18 (0.011)			
Reaction times (ms)						
Non‐depressed PD						
Repeat	510.4 (24.6)	506.6 (26.3)	3.8 (4.7)	558.5 (25.4)	544.1 (25.0)	14.4 (4.4)
Switch	530.4 (30.2)	514.4 (28.3)	16.1 (5.7)	570.0 (28.0)	563.7 (29.5)	6.3 (5.3)
PD with depression (history)						
Repeat	563.7 (27.6)	547.7 (27.0)	16.0 (4.3)	558.3 (33.5)	540.6 (32.8)	17.7 (3.9)
Switch	574.9 (30.8)	571.8 (32.1)	3.1 (4.8)	565.6 (34.2)	564.9 (38.6)	0.6 (5.7)
Healthy controls						
Repeat	505.4 (24.1)	500.4 (24.2)	5.0 (4.5)			
Switch	520.6 (26.8)	519.8 (26.0)	0.8 (5.2)			

Values represent mean proportion of incorrect responses in % and mean response times in ms (standard errors of the mean). PD, Parkinson's disease.

First, we assessed whether there were any drug effects in PD patients by means of an analysis of variance (ANOVA) with the within‐subject factors DRUG (ON, OFF), REWARD (high, low) and TRIAL TYPE (switch, repeat). Subsequently, in the absence of a medication effect, we averaged patient's error rates and reaction times across the two drug sessions and compared these measurements with measurements obtained in healthy controls by means of a mixed ANOVA with REWARD (high, low) and TRIAL TYPE (switch, repeat) as within‐subject factors and GROUP (non‐depressed PD, depressed PD and healthy control) as a between‐subject factor. When the overall mixed ANOVA revealed a significant interaction with GROUP as a factor, we performed subsequent mixed ANOVA's to compare the GROUPS separately breaking‐down this interaction. In these cases, the factor GROUP comprises only two levels. For the overall interaction we use “GROUP_(3)_” (referring to three within‐subject levels) and when breaking‐down the overall interaction comparing two groups, we use “GROUP_(2)_” (referring to two within‐subject levels). Statistical inference was set at a threshold of *p *<* *0.05. Partial Eta squared ($\eta_{p}^{2}$) is reported as measure of effect size.

## 3 RESULTS

### 3.1 Effect of reward motivation on task‐switching

#### 3.1.1 Error rates

In PD patients, there was no significant main effect of DRUG and none of the interaction effects with DRUG as a factor was significant (Supporting information Table S1). Therefore, we averaged patient's error rates across the two drug sessions and compared these measurements with measurements obtained in healthy controls.

There was no main effect of REWARD (*F*_2,65_ = 0.71, *p *=* *0.40, $\eta_{p}^{2}$ = 0.011). We did observe a significant main effect of TRIAL TYPE (*F*_2,65_ = 45.18, *p *<* *0.001, $\eta_{p}^{2}$ = 0.410), indicating that all subjects made more errors on switch compared with repeat trials. This was not different between PD patients (depressed and non‐depressed) and healthy controls (TRIAL‐TYPE × GROUP_(3)_: *F*_2,65_ = 0.61, *p *=* *0.55, $\eta_{p}^{2}$ = 0.018).

**Figure 1:**
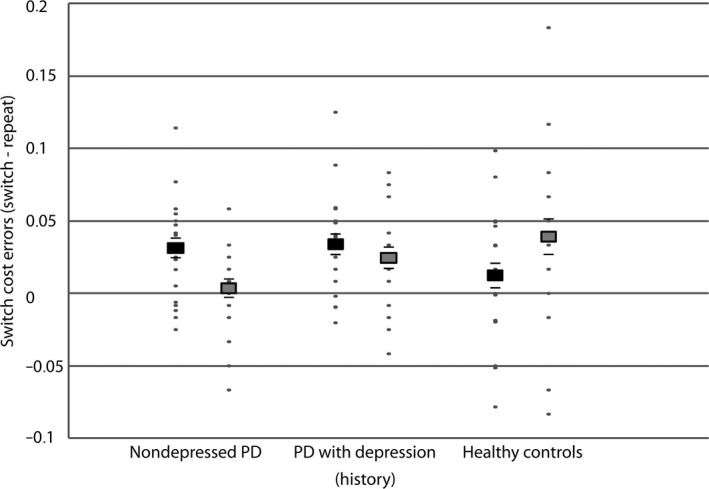
Effects of reward motivation on task‐switching. Average switch cost [switch repeat] on error rates on low‐ (black squares) and high‐reward trials (grey squares) in non‐depressed PD patients, PD patients with a depression (history) and healthy controls. Black lines represent the (positive and negative) standard error of the mean. Individual data points are plotted in grey dots. PD, Parkinson's disease

Consistent with our previous study (Aarts et al., 2011), we observed that PD patients exhibited greater beneficial effects of reward motivation on task‐switching than healthy controls evidenced by a significant three‐way interaction among TRIAL‐TYPE, REWARD and GROUP_(3)_ (*F*_2,65_ = 5.11, *p *=* *0.009, $\eta_{p}^{2}$ ^^= 0.136) (Figure 1). This beneficial effect of reward motivation on task‐switching did not differ between PD patients with and without depression (history), evidenced by a non‐significant TRIAL‐TYPE × REWARD × GROUP_(2)_ interaction when comparing PD patients with and without depression (history) (*F*_1,43_ = 0.62, *p *=* *0.43, $\eta_{p}^{2}$ = 0.014). Both PD groups showed a greater beneficial effect of reward motivation on task‐switching than healthy controls (comparison of non‐depressed PD with healthy controls, *F*_1,44_ = 7.84, *p *=* *0.008, $\eta_{p}^{2}$ = 0.151; comparison of depressed PD patients with healthy controls, *F*_1,43_ = 4.76, *p *=* *0.035, $\eta_{p}^{2}$ = 0.100). Breakdown of this interaction revealed greater beneficial effects of reward motivation on switch trials in patients (from both patient groups) than in healthy controls (REWARD × GROUP_(3)_ interaction on switch trials, *F*_2,65_ = 5.22, *p *=* *0.008, $\eta_{p}^{2}$ = 0.138; comparison of non‐depressed PD with healthy controls, *F*_1,44_ = 9.91, *p *=* *0.003, $\eta_{p}^{2}$ = 0.184; comparison of PD patients with a depression (history) and healthy controls, *F*_1,43_ = 4.62, *p *=* *0.037, $\eta_{p}^{2}$ = 0.097). No such interaction was observed for repeat trials (REWARD × GROUP_(3)_ interaction on repeat trials, *F*_2,65_ = 0.64, *p *=* *0.53, $\eta_{p}^{2}$ = 0.019). Post hoc paired samples *t* tests revealed that non‐depressed PD patients made significantly fewer errors on high‐reward switch trials compared with low‐reward switch trials (*t*_(22)_ = 2.90, *p *=* *0.008, *d *=* *0.618). No such effect was observed in depressed PD patients (*t*_(21)_ = 1.24, *p *=* *0.23, *d *=* *0.259). Conversely, healthy, age‐matched controls tended to make more errors on high‐reward switch trials compared with low‐reward switch trials (*t*_(22)_ = −1.79, *p *=* *0.087, *d *=* *−0.373). There was no TRIAL‐TYPE × GROUP_(3)_ interaction for low‐reward trials (*F*_2,65_ = 0.887, *p *=* *0.42, $\eta_{p}^{2}$ = 0.027), but on high‐reward trials, switch costs were lower for PD patients than healthy controls (TRIAL‐TYPE × GROUP_(3)_: *F*_2,65_ = 3.34, *p *=* *0.042, $\eta_{p}^{2}$ = 0.093).

Previously, we have shown that effects of reward motivation are more readily observed on the most established task set (i.e. the arrow task) (Aarts et al., 2011). Therefore, we performed an extra analysis including TASK (i.e. arrow or word) as additional within‐subject factor. In patients, in terms of error rates, there was no significant DRUG × REWARD × TRIAL‐TYPE × TASK × GROUP_(2)_ interaction (*F*_1,43_ = 1.88, *p* = 0.18, $\eta_{p}^{2}$ = 0.042) and no significant DRUG × REWARD × TRIAL‐TYPE × TASK interaction (*F*_1,43_ = 0.11, *p* = 0.74, $\eta_{p}^{2}$ = 0.003). Therefore, to compare patient data with healthy control data, we averaged patient data across drug sessions. Comparison of depressed patients, non‐depressed patients and healthy controls revealed no differences in terms of the effect of task (i.e. arrow or word) on reward motivated cognitive control, indicated by a non‐significant interaction among REWARD, TRIAL‐TYPE, TASK and GROUP_(3)_ (*F*_2,65_ = 0.01, *p* = 0.99, $\eta_{p}^{2}$ = 0.000).

To assess whether the beneficial effect of reward motivation on task‐switching in PD patients varied as a function of current depression severity, we correlated the effect of reward motivation (low–high reward) on switch cost (switch repeat) with the BDI score during the OFF session. This analysis revealed that reward motivational enhancement of task‐switching in the PD group as a whole did not vary as a function of current depression severity (*r*_(45)_ = 0.152, *p *=* *0.32). Additional subgroup analyses (comparison of currently depressed patients with never depressed patients and of patients who suffer(ed) from mild depressive symptoms with patients who suffer(ed) from a major depressive episode) can be found in the supplement.

#### 3.1.2 Reaction times

There was no main effect of DRUG and no significant interactions with DRUG as a factor in PD patients (Supporting information Table S1). There was a near significant GROUP_(2)_ by DRUG interaction (*F*_1,43_ = 4.00*, p *=* *0.052*,* $\eta_{p}^{2}$ = 0.085). However, breakdown of this interaction revealed no significant drug effect on reaction times in neither PD group (non‐depressed PD *p* = 0.07, depressed PD *p* = 0.47). Moreover, all interactions of interest with drug as a factor were not significant. Therefore, we averaged patient's reaction times across the two drug sessions and compared these measurements with measurements obtained in healthy controls.

Comparison of PD patients with and without a depression (history) and healthy controls revealed a significant main effect of REWARD (*F*_2,65_ = 20.55, *p *<* *0.001, $\eta_{p}^{2}$ = 0.240), indicating faster reaction times on high‐ versus low‐reward trials across all three groups. There was also a significant main effect of TRIAL‐TYPE (*F*_1,65_ = 45.01, *p *<* *0.001, $\eta_{p}^{2}$ = 0.409), indicating slower reaction times on the more demanding switch than repeat trials in all three groups. There were no significant interactions with GROUP_(3)_ as a factor, indicating that PD patients with and without depression (history) and healthy controls did not differ in terms of reaction times (Supporting information Table S2).

Again, we performed an extra analysis including TASK (i.e. arrow or word) as additional within‐subject factor. In patients, in terms of reaction times, there was no significant DRUG × REWARD × TRIAL‐TYPE × TASK × GROUP_(2)_ interaction (*F*_1,43_ = 0.73, *p* = 0.40, $\eta_{p}^{2}$ = 0.017) and no significant DRUG × REWARD × TRIAL‐TYPE × TASK interaction (*F*_1,43_ = 1.47, *p* = 0.23, $\eta_{p}^{2}$ = 0.033). Therefore, to compare patient data with healthy control data, we averaged patient data across drug sessions. Comparison of depressed patients, non‐depressed patients and healthy controls revealed no differences in terms of the effect of task (i.e. arrow or word) on reward motivated cognitive control, indicated by a non‐significant interaction between REWARD, TRIAL‐TYPE, TASK and GROUP_(3)_ (*F*_2,65_ = 0.00, *p* = 0.99, $\eta_{p}^{2}$ = 0.000).

There were no significant correlations between behavioural findings in terms of reaction times and current depression severity. Again, additional subgroup analyses can be found in the supplement.

In sum, PD patients with and without depression (history) showed reward motivational enhancement of task‐switching in terms of error rates, whereas reward motivation, if anything, impaired task‐switching in matched healthy controls. We did not observe any differences between PD with and without depression (history) patients and healthy controls in terms of reaction times and there were no effects of medication.

## 4 DISCUSSION

This study replicates previous findings by demonstrating reward motivational enhancement of task‐switching in terms of error rates in PD patients compared with healthy controls (Aarts et al., 2011). In addition, we extend previous findings by showing that reward motivational enhancement of task‐switching in PD is unaltered by depression (history).

The finding that reward motivation has contrasting effects on task‐switching in patients with PD and healthy controls is a direct replication of our previous study with the exact same paradigm (Aarts et al., 2011) and strengthens our belief in the observation that PD is accompanied by enhanced beneficial impact of incentive on cognitive control. The hypothesis that the potentiation of cognitive motivation in PD patients, observed here and in our previous study, reflects dopamine deficiency is supported by the observation in that previous study that effects correlated with dopamine cell loss in dorsal striatum. Moreover, it is also strengthened by our previous study in healthy volunteers, in which higher baseline dopamine synthesis capacity was associated with greater detrimental effects of reward motivation on cognitive control (Aarts, Wallace, et al., 2014).

One potential caveat that we considered is the possibility that there was more dynamic range for reward motivation to impact performance in PD patients than in controls. In other words, it is easier to potentiate performance if it is impaired to begin with. However, this was not the case. There was no task‐switching deficit in PD patients compared with controls in the low‐reward trials. Therefore, we can exclude that potential confound. Of course this observation does raise a different question: Why did the present study not reveal a task‐switching deficit in PD, as did previous studies (Downes et al., 1989; Cools et al., 2001a,b, 2003; Pollux, 2004; Shook, Franz, Higginson, Wheelock, & Sigvardt, 2005; Witt et al., 2006), but see (Kehagia, Cools, Barker, & Robbins, 2009)? One possibility is that the current task was not optimized for detecting such task‐switching deficits (Kehagia et al., 2009), although we have previously used the exact same task to demonstrate subtle but significant deficits (Aarts et al., 2011; Aarts, Nusselein, et al., 2014). More likely is the possibility that any task‐switching deficit was remedied by the reward context in which trials were presented. While the presence of an incentive potentiated performance disproportionally on the switch trials, it is possible that any benefit generalized to the repeat trials (relative to a non‐rewarded context).

A striking feature of our current and previous data is that, in age‐matched healthy controls, reward motivation tended to impair rather than enhance task‐switching. As such, the age‐matched control group resembled if anything the younger volunteers from the prior study with higher striatal dopamine synthesis capacity (Aarts, Wallace, et al., 2014). This is in line with recent reports that ageing is accompanied by upregulated striatal dopamine synthesis capacity (Berry et al., 2016).

One aspect of our results that is surprising in the context of the previous PET and SPECT studies using this paradigm is the failure to find an effect of dopaminergic medication. There was no effect of dopaminergic medication on task‐switching or on the interaction between reward motivation and task‐switching in our PD patients. The absence of a main effect of dopaminergic medication on task‐switching is particularly unexpected given prior evidence for beneficial effects of dopaminergic medication on task‐switching in PD (Hayes, Davidson, Keele, & Rafal, 1998; Cools et al., 2001a, 2003; Shook et al., 2005; Kehagia et al., 2009). Moreover, the association between the incentivization of cognitive control and indices of striatal dopamine transmission in previous studies (Aarts et al., 2011; Aarts, Wallace, et al., 2014) renders the absence of an effect of dopaminergic medication on this task remarkable. After all, we would have expected any dopamine dependency to surface in terms of an effect of medication withdrawal. We remain puzzled about this lack of effects, and speculate that this reflects either a suboptimal withdrawal procedure (given that the used compounds have long half‐lives) or individual genetic differences, as shown previously in a study with the same paradigm to assess dopaminergic drug effects in ADHD (van Holstein et al., 2011; Aarts et al., 2015).

PD patients with depression (history) showed similar reward motivational enhancement of task‐switching as did non‐depressed PD patients. Thus, the beneficial effect of reward motivation on cognitive control is potentiated even in (previously) depressed PD patients. This might be surprising given decreased reward motivation in depression (Henriques & Davidson, 2000; Knutson et al., 2008; Stoy et al., 2012; Yang, Sajatovic, & Walter, 2012; Treadway & Zald, 2013; Yang et al., 2016). One potential caveat of this study is the heterogeneity of the patient group with depression (history): This group included patients with past and present depression and sample sizes of the subgroups were too small to make meaningful direct comparisons between groups with current and past depression. As such, we can only conclude that a depression history in Parkinson's disease does not alter reward motivational enhancement of cognitive control. The question whether a current depression diminishes incentive motivation of cognitive control should be addressed in future work.

The finding of enhanced incentive motivation of cognitive control replicated here is perhaps reminiscent of the phenomenon of ‘paradoxical kinesia’. Nevertheless, it contrasts with previous studies showing reduced motivation for physical effort in PD (Porat et al., 2014; Chong et al., 2015; Le Bouc et al., 2016), although these have focused primarily on intrinsic motivation (value‐based choice to exert effort). Studies investigating extrinsic (incentive) motivation for physical effort in PD are still scarce, but suggest similar effects (Le Bouc et al., 2016). It will be interesting to compare, in future work, the effect of PD on extrinsic and intrinsic motor and cognitive motivation.

Which mechanism might underlie the effect of PD on incentivizing cognitive control? One possibility, inspired by opportunity cost accounts of tonic dopamine's role in motivating vigour (physical effort) (Niv et al., 2007) as well as cognitive control (Kurzban et al., 2013; Boureau, Sokol‐Hessner, & Daw, 2015), is that increases in tonic dopamine might correspond to increases in a net average reward rate of the environment against which rewards are compared. A separate, but relevant line of evidence concerns the phenomenon of adaptive coding of reward (Tobler, Fiorillo, & Schultz, 2005; Louie, Glimcher, & Webb, 2015) and studies indicating that a potential reward is subjectively more valuable in a reward‐poor environment (Stewart, Chater, Stott, & Reimers, 2003; Louie, Khaw, & Glimcher, 2013; Rigoli, Friston, & Dolan, 2016; Rigoli, Rutledge, Dayan, & Dolan, 2016). As such, one possible explanation of the present findings is that PD patients, whose tonic dopamine level and putative corresponding average reward rate are excessively low, evaluate a reward as relatively more valuable than healthy controls, making them more likely to engage in a high‐demand task when a reward is at stake. To test this hypothesis, instantaneous and average reward rate should be manipulated in an orthogonal manner, as has been done previously for testing the dopamine‐dependent opportunity cost account of physical as well as cognitive vigour (Guitart‐Masip, Beierholm, Dolan, Duzel, & Dayan, 2011; Beierholm et al., 2013; Otto & Daw, 2018).

In sum, we replicated findings of reward motivational enhancement of cognitive control in PD patients and extended these findings to PD patients with concurrent depression. These findings suggest that PD patients might benefit from incentive motivational strategies to overcome their cognitive deficits associated with the disease.

## CONFLICT OF INTEREST

The authors declare no competing financial interests.

## DATA ACCESSIBILITY

Data are available via request from the authors.

## AUTHOR CONTRIBUTIONS

MT and RC drafted and revised the report. MT was responsible for the data collection and analyses of the data. All authors were responsible for the conception and design of the study. All authors approved the final version of the manuscript.
