# Supplementary material for: Enhanced motivation of cognitive control in Parkinson's disease
Source: Eur J Neurosci. 2018 Sep 16;48(6):2374–84. doi: 10.1111/ejn.14137 (PMC6175070; doi:10.1111/ejn.14137)
Supplement: Supplementary file 2 [file EJN-48-2374-s002.docx]

**Supplement**

**Table S1. Drug effects in patients with Parkinson’s disease**

| **Error rates** |  |
| --- | --- |
| DRUG  DRUG x GROUP  DRUG x REWARD  DRUG x REWARD x GROUP  DRUG x TRIAL-TYPE  DRUG x TRIAL-TYPE x GROUP  DRUG x REWARD x TRIAL-TYPE  DRUG x REWARD x TRIAL-TYPE x GROUP | *F*_(1,43)_=3.27, *p*=0.078, ηp^2^=0.071  *F*_(1,43)_=2.66, *p*=0.110, ηp^2^=0.058  *F*_(1,43)_=0.00, *p*=0.973, ηp^2^=0.000  *F*_(1,43)_=0.46, *p*=0.502, ηp^2^=0.011  *F*_(1,43)_=0.51, *p*=0.823, ηp^2^=0.001  *F*_(1,43)_=0.46, *p*=0.503, ηp^2^=0.011  *F*_(1,43)_=1.01, *p*=0.322, ηp^2^=0.023  *F*_(1,43)_=0.07, *p*=0.789, ηp^2^=0.002 |
| **Reaction times** |  |
| DRUG  DRUG x GROUP  DRUG x REWARD  DRUG x REWARD x GROUP  DRUG x TRIAL-TYPE  DRUG x TRIAL-TYPE x GROUP  DRUG x REWARD x TRIAL-TYPE  DRUG x REWARD x TRIAL-TYPE x GROUP | *F*_(1,43)_=1.46, *p*=0.234, ηp^2^=0.033  *F*_(1,43)_=4.00, *p*=0.052, ηp^2^=0.085  *F*_(1,43)_=0.10, *p*=0.759, ηp^2^=0.002  *F*_(1,43)_=0.01, *p*=0.913, ηp^2^=0.000  *F*_(1,43)_=0.01, *p*=0.921, ηp^2^=0.000  *F*_(1,43)_=0.26, *p*=0.615, ηp^2^=0.006  *F*_(1,43)_=2.47, *p*=0.123, ηp^2^=0.054  *F*_(1,43)_=2.14, *p*=0.151, ηp^2^=0.047 |

**Table S2. Analysis of reaction times in PD patients with and without a depression (history) and controls**

| GROUP  REWARD x GROUP  TRIAL-TYPE x GROUP  REWARD x TRIAL-TYPE x GROUP | *F*_(2,65)_=0.87, *p*=0.422, ηp^2^=0.026  *F*_(2,65)_=2.13, *p*=0.127, ηp^2^=0.062  *F*_(2,65)_=0.48, *p*=0.623, ηp^2^=0.014  *F*_(1,43)_=1.26, *p*=0.291, ηp^2^=0.037 |
| --- | --- |

PD = Parkinson’s disease

**Subgroup analysis**

We performed subgroup analyses comparing error rates in currently depressed patients (n=7) with error rates in never depressed patients (i.e. the nondepressed patient group). This comparison revealed no differences, evidenced by a non-significant 3-way interaction between TRIAL-TYPE, REWARD and GROUP (F(1,28)=0.58, p=0.45, ηp2=0.02).

However, when comparing reaction times between patients with a current depression and never depressed patients, we did observe a significant 3-way interaction between TRIAL-TYPE, REWARD and GROUP (F(1,28)=0.7.19, p=0.012, ηp2=0.204)). Break-down of this interaction revealed a significant TRIAL-TYPE x REWARD interaction in the currently depressed patients (F(6)=14.36, p=0.012, ηp2=0.705)), but not in the never depressed patients (F(22)=0.07, p=0.80, ηp2=0.03)). Post-hoc paired samples t-tests in currently depressed patients revealed that reward significantly decreased reaction times on repeat trials (t(6)=3.86, p=0.008, d=0.17). No such effect was observed on switch trials (t(6)=0.02, p=0.98, d=0.00).

We also performed subgroup analyses comparing patients who suffer(ed) from mild depressive symptoms (n=15) with patients who suffer(ed) from a major depressive episode (n=7). These analyses revealed no significant differences in terms of error rates (non-significant 3-way interaction between TRIAL-TYPE, REWARD and GROUP (F(1,20)=0.75, p=0.40, ηp2=0.04)) and no significant differences in terms of reaction times (non-significant 3-way interaction between TRIAL-TYPE, REWARD and GROUP (F(1,20)=0.00, p=0.98, ηp2=0.00).
