# Supplementary material for: Enhanced motivation of cognitive control in Parkinson's disease
Source: Eur J Neurosci. 2018 Sep 16;48(6):2374–84. doi: 10.1111/ejn.14137 (PMC6175070; doi:10.1111/ejn.14137)
Supplement: Supplementary file 1 [file EJN-48-2374-s001.docx]

Enhanced motivation of cognitive control in Parkinson’s disease

Timmer Monique; Aarts, Esther; Esselink, Rianne; Cools, Roshan

_____________________________________________________________________________________

| Review timeline: | Submission date: 05/01/18  Editorial Decision: Rejection with resubmission  Revision Received: 12/07/18  Editorial Decision: Minor Revision  Revision Received: 17/08/18  Accepted: 20/08/18 |  |
| --- | --- | --- |
|  |  |  |

Editor: Yoland Smith

Reviewer 1: Felicia Goldstein

Reviewer 2: Christelle Baunez

_____________________________________________________________________________________

1st Editorial Decision	05/03/18

_____________________________________________________________________________________

Enhanced motivation for cognitive control in Parkinson’s disease

Dear Dr. Timmer,

First of all, apologies for the time it has taken to deal with your manuscript.  It has now been reviewed by external reviewers as well as by the Section Editor, Dr. Yoland Smith, and ourselves.  Based on these reviews, we regret to inform you that we are not able to accept your manuscript for publication in EJN in its present form. However, the research described in your manuscript is potentially of interest and therefore we invite you to resubmit a much revised version.

As you can see, both of the reviewers consider the topic under study of high relevance and that your manuscript is well written. However, they both raised a major concern about the study design and the choice of patients for this study. Patients with current vs past depression should have been categorized separately. In its present form, the pool of patients is too heterogeneous, which complicates interpretation of the data gathered from these individuals.  A comparative analysis of patients with current vs past depression should be provided.  Please carefully address these points in your resubmitted version.  Please also define 'SPECT'', provide a list of abbreviations and carefully check the reference list for missing information.  

When revising the manuscript, please embolden or underline major changes to the text so they are easily identifiable and DO NOT leave 'track change' formatting marks in your paper. If the changes made are extensive, please also provide an unmarked version.

Please provide a detailed response to the reviewers' comments, describing the changes you have made in the text. Please upload this response letter as a separate Word (.doc) file using the file designation "Authors' Response to Reviewers" when uploading your manuscript files.

When finalized, please upload your complete revised manuscript onto the website as a Word (.doc or .docx), or .rtf file.  Please also ensure that a complete set of tables and figures is included as separate files, even if these have not changed from the originals.  At this stage it is necessary to provide high resolution figures.  Please see important instructions below.

To re-submit your manuscript, please go to your Author Centre and check in 'Manuscripts with decisions' under the previous manuscript number for a 'Create a resubmission' link.  Also, please note that only the Author who submitted the original version of the manuscript should submit a revised version.

If you feel that your paper could benefit from English language polishing, you may wish to consider having your paper professionally edited for English language by a service such as Wiley’s at http://wileyeditingservices.com. Please note that while this service will greatly improve the readability of your paper, it does not guarantee acceptance of your paper by the journal.

Thank you for submitting your work to EJN.

Kind regards,

Paul Bolam & John Foxe
co-Editors in Chief, EJN


Reviews:

Reviewer: 1

Comments to the Author
This is a well-written paper and a carefully conducted study that replicates the authors' prior findings of enhanced incentive motivation for cognitive control in patients with Parkinson's disease.  My main criticism involves the evaluation of the influence of depression on reduced sensitivity.  I do not think the authors have adequately tested this in their study.  A stronger test would be to examine patients with current rather than past depression.  Although the BDI scores are higher in the patients with a history, the scores are not clinically significantly elevated.  Did the authors consider looking at the performance separately of the seven patients with current depression to see if there is any difference in performance from the never depressed group?  I think the authors need to suggest a further test of their hypothesis and to eliminate or tone down the conclusion that "depression-related motivation deficit is not sufficiently large to overrule the dorsal striatal depletion-related potentiation....." on page 16.

Reviewer: 2

Comments to the Author
In this manuscript, the authors replicate a former study, assessing the effects of medication of two groups of parkinsonian patients (non-depressed and with a history of depression PD) in a cognitive task previously used by this group. The main findings were previously shown and the new aspect (depressed non-depressed PD) is weakened by the fact these patients are heterogeneous and should have been subdivided in terms of past depression vs current depression. Severity of depression has been taken into account but only at the time of testing, maybe the severity at the peak moment of depression for those who are no longer depressed should be integrated.
other points:
in the abstract: "relatively OFF DA medication" is weird. Since the authors consider the patients OFF medication and they explain it in the methodology, they should remove "relatively"
As previously said, the fact that the PD depressed group is not homogeneous is problematic. Some are under treatment, which can introduce a bias. The variability in severity of depression and history is also problematic: some are currently depressed, others have a recent history and others have a past history. This should be analyzed in further details, especially since it is the novel part of the study.
Statistical analyses: I am not sure that it is allowed to perform the analyses between only two groups sometimes, between the 3 groups in other cases, especially if there is no significant global interaction.
Page12 : statistics for RT: it is not clear to which groups 'Group' refers to. Is it all PD pooled together or the depressed PD vs non depressed and healthy controls?
Could the type of switch (word to arrow vs arrow to word) be influenced in a different manner? Did the authors look at this?

------------------------

____________________________________________________________________________________

Authors’ Response	12/07/2018

_____________________________________________________________________________________

**Rebuttal letter**

Ref.: EJN-2018-01-25237

Enhanced motivation for cognitive control in Parkinson’s disease

European Journal of Neuroscience

We thank the editor for the invitation to revise and resubmit our paper to the European Journal of Neuroscience. We are particularly grateful for the time and care that the reviewers and editor put into reading and commenting on our paper. We found these reviews to be constructive and believe that addressing the comments has significantly improved the paper. Please find each comment below, followed by our responses in italic font with our actions underlined. Specific changes to the text are quoted in indented text, and are also highlighted in yellow in the revised manuscript. We very much hope that you now consider our paper suitable for publication in the European Journal of Neuroscience.

**Reviewer #1 (comments to the author):**

This is a well-written paper and a carefully conducted study that replicates the authors' prior findings of enhanced incentive motivation for cognitive control in patients with Parkinson's disease.  My main criticism involves the evaluation of the influence of depression on reduced sensitivity. I do not think the authors have adequately tested this in their study.  A stronger test would be to examine patients with current rather than past depression. Although the BDI scores are higher in the patients with a history, the scores are not clinically significantly elevated. Did the authors consider looking at the performance separately of the seven patients with current depression to see if there is any difference in performance from the never depressed group? I think the authors need to suggest a further test of their hypothesis and to eliminate or tone down the conclusion that "depression-related motivation deficit is not sufficiently large to overrule the dorsal striatal depletion-related potentiation....." on page 16.

*AU: We thank the reviewer for this comment and now provide an additional analysis comparing the currently depressed Parkinson’s disease patients with the never depressed patients. This analysis revealed no significant differences in terms of error rates between currently depressed patients and never depressed patients (non-significant 3-way interaction between TRIAL-TYPE, REWARD and GROUP (F(1,28)=0.58, p=0.45, ηp2=0.02)). When comparing reaction times between patients with a current depression and never depressed patients, we did observe a significant 3-way interaction between TRIAL-TYPE, REWARD and GROUP (F(1,28)=0.7.19, p=0.012, ηp2=0.204)). Break-down of this interaction revealed a significant TRIAL-TYPE x REWARD interaction in the currently depressed patients (F(6)=14.36, p=0.012, ηp2=0.705)), but not in the never depressed patients (F(22)=0.07, p=0.80, ηp2=0.03)). Post-hoc paired samples t-tests in currently depressed patients revealed that reward significantly decreased reaction times on repeat trials (t_(6)_=3.86, p=0.008, d=0.17), but not on switch trials (t_(6)_=0.02, p=0.98, d=0.00), thus in fact enhancing the RT switch cost.*

*However, we believe that these findings should be interpreted with caution as the currently depressed group only comprised seven patients. Therefore, we decided to report these subgroup analyses in the supplementary materials and not in the main paper. We agree with the reviewer that the present findings do not provide a definite answer to the question whether or not currently depressed patients exhibit diminished incentive motivation for cognitive control and have toned down our conclusion in the discussion section.*

*We have now added the following sentences to the supplement:*

*“We performed subgroup analyses comparing error rates in currently depressed patients (n=7) with error rates in never depressed patients (i.e. the nondepressed patient group). This comparison revealed no differences, evidenced by a non-significant 3-way interaction between TRIAL-TYPE, REWARD and GROUP (F(1,28)=0.58, p=0.45, ηp2=0.02).*

*However, when comparing reaction times between patients with a current depression and never depressed patients, we did observe a significant 3-way interaction between TRIAL-TYPE, REWARD and GROUP (F(1,28)=0.7.19, p=0.012, ηp2=0.204)). Break-down of this interaction revealed a significant TRIAL-TYPE x REWARD interaction in the currently depressed patients (F(6)=14.36, p=0.012, ηp2=0.705)), but not in the never depressed patients (F(22)=0.07, p=0.80, ηp2=0.03)). Post-hoc paired samples t-tests in currently depressed patients revealed that reward significantly decreased reaction times on repeat trials (t_(6)_=3.86, p=0.008, d=0.17). No such effect was observed on switch trials (t_(6)_=0.02, p=0.98, d=0.00).”*

*And the following sentences to the main paper:*

*“Additional subgroup analyses (comparison of currently depressed patients with never depressed patients and of patients who suffer(ed) from mild depressive symptoms with patients who suffer(ed) from a major depressive episode) can be found in the supplement.”*

*Moreover, the following sentences of the discussion section...:*

*“One potential caveat of the present study is the heterogeneity of the patient group with depression (history): This group included patients with past and present depression. However, there are two reasons for thinking that the absence of an effect of depression does not reflect this heterogeneity. First, reward motivational deficits in patients with depression have been shown to extend to individuals who are currently not depressed but who are at risk of depression (Olino et al., 2014), but see (Yang et al., 2014). Second, in the present study, behavioral effects did not vary as a function of current depression severity. Although we do not exclude the possibility that more severely affected depressed PD patients will exhibit reduced motivational enhancement of cognitive control, we now speculate that the depression-related motivation deficit is not sufficiently large to overrule the dorsal striatal depletion-related potentiation of motivated cognition common in both depressed and nondepressed PD patients.”*

*… have been replaced by:*

*“One potential caveat of the present study is the heterogeneity of the patient group with depression (history): This group included patients with past and present depression and sample sizes of the subgroups were too small to make meaningful direct comparisons between groups with current and past depression. As such, we can only conclude that a depression history in Parkinson’s disease does not alter reward motivational enhancement for cognitive control. The question whether a current depression diminishes incentive motivation for cognitive control should be addressed in future work.”*

**Reviewer #2 (comments to the author):**
In this manuscript, the authors replicate a former study, assessing the effects of medication of two groups of parkinsonian patients (non-depressed and with a history of depression PD) in a cognitive task previously used by this group. The main findings were previously shown and the new aspect (depressed non-depressed PD) is weakened by the fact these patients are heterogeneous and should have been subdivided in terms of past depression vs current depression. Severity of depression has been taken into account but only at the time of testing, maybe the severity at the peak moment of depression for those who are no longer depressed should be integrated.

*AU: We thank the reviewer for this comment and would also like to refer to the response provided above to the comment of reviewer 1. We now provide an additional analysis comparing the Parkinson’s disease patients with a current depression (n=7) with the never depressed patients (n=23). This analysis revealed no significant differences in terms of error rates between currently depressed patients and never depressed patients (non-significant 3-way interaction between TRIAL-TYPE, REWARD and GROUP (F(1,28)=0.58, p=0.45, ηp2=0.02)). In terms of reaction times, we did observe a significant 3-way interaction between TRIAL-TYPE, REWARD and GROUP (F(1,28)=0.7.19, p=0.012, ηp2=0.204)). Break-down of this interaction revealed a significant TRIAL-TYPE x REWARD interaction in the currently depressed patients (F(6)=14.36, p=0.012, ηp2=0.705)), but not in the never depressed patients (F(22)=0.07, p=0.80, ηp2=0.03)). Post-hoc paired samples t-tests in currently depressed patients revealed that reward significantly decreased reaction times on repeat trials (t_(6)_=3.86, p=0.008, d=0.17), but not on switch trials (t_(6)_=0.02, p=0.98, d=0.00).*

*Moreover, as suggested, we also performed an additional analysis comparing patients who suffer(ed) from only mild depressive symptoms (n=15) with patients who suffer(ed) from a major depressive episode (n=7). This analysis revealed no significant differences in terms of error rates, evidenced by a non-significant 3-way interaction between TRIAL-TYPE, REWARD and GROUP (F(1,20)=0.75, p=0.40, ηp2=0.04). There was also no significant difference in terms of reaction times (F(1,20)=0.00, p=0.98, ηp2=0.00).*

*We believe that the results of these subgroup analyses should be interpreted with caution, because the subgroups comprised only very few patients. Therefore, we decided to report these analysis in the supplementary materials and not in the main paper.*

*We have now added the following sentences to the supplement:*

*“We performed subgroup analyses comparing error rates in currently depressed patients (n=7) with error rates in never depressed patients (i.e. the nondepressed patient group). This comparison revealed no differences, evidenced by a non-significant 3-way interaction between TRIAL-TYPE, REWARD and GROUP (F(1,28)=0.58, p=0.45, ηp2=0.02).*

*However, when comparing reaction times between patients with a current depression and never depressed patients, we did observe a significant 3-way interaction between TRIAL-TYPE, REWARD and GROUP (F(1,28)=0.7.19, p=0.012, ηp2=0.204)). Break-down of this interaction revealed a significant TRIAL-TYPE x REWARD interaction in the currently depressed patients (F(6)=14.36, p=0.012, ηp2=0.705)), but not in the never depressed patients (F(22)=0.07, p=0.80, ηp2=0.03)). Post-hoc paired samples t-tests in currently depressed patients revealed that reward significantly decreased reaction times on repeat trials (t_(6)_=3.86, p=0.008, d=0.17). No such effect was observed on switch trials (t_(6)_=0.02, p=0.98, d=0.00).”*

*“We also performed subgroup analyses comparing patients who suffer(ed) from mild depressive symptoms (n=15) with patients who suffer(ed) from a major depressive episode (n=7). These analyses revealed no significant differences in terms of error rates (non-significant 3-way interaction between TRIAL-TYPE, REWARD and GROUP (F(1,20)=0.75, p=0.40, ηp2=0.04)) and no significant differences in terms of reaction times (non-significant 3-way interaction between TRIAL-TYPE, REWARD and GROUP (F(1,20)=0.00, p=0.98, ηp2=0.00).*

*Moreover, we have added the following sentences to the main paper:*

*“Additional subgroup analyses (comparison of currently depressed patients with never depressed patients and of patients who suffer(ed) from mild depressive symptoms with patients who suffer(ed) from a major depressive episode) can be found in the supplement.”*

Other points:
In the abstract: "relatively OFF DA medication" is weird. Since the authors consider the patients OFF medication and they explain it in the methodology, they should remove "relatively".

*AU: We have removed “relatively” in the abstract.*

As previously said, the fact that the PD depressed group is not homogeneous is problematic. Some are under treatment, which can introduce a bias. The variability in severity of depression and history is also problematic: some are currently depressed, others have a recent history and others have a past history. This should be analyzed in further details, especially since it is the novel part of the study.

*AU: We thank the reviewer for pointing this out and would also like to refer to our response above. Besides the correlation analysis (correlating the effect of reward motivation (low-high reward) on switch-cost (switch-repeat) with the BDI score during the OFF session) that was already provided in the main text, we have now added two additional analyses to the supplementary materials: One comparing currently depressed patients with never depressed patients and one comparing patients who suffer(ed) from mild depressive symptoms with patients who suffer(ed) from a major depressive episode.*

*Moreover, we have toned down our conclusion in the discussion section.*

*“As such, we can only conclude that a depression history in Parkinson’s disease does not alter reward motivational enhancement for cognitive control. However, the question whether a current depression diminishes incentive motivation for cognitive control should be addressed in future work.”*

Statistical analyses: I am not sure that it is allowed to perform the analyses between only two groups sometimes, between the 3 groups in other cases, especially if there is no significant global interaction.

Page12 : statistics for RT: it is not clear to which groups 'Group' refers to. Is it all PD pooled together or the depressed PD vs non depressed and healthy controls?

*AU: We thank the reviewer for this comment and understand the confusion. We would like to point out that all analyses conducted start with a mixed ANOVA with 2 within-subject levels (REWARD; high and low, and TRIAL-TYPE; switch and repeat) and 1 between-subject level (GROUP; nondepressed PD, depressed PD and healthy control). Only significant interactions with GROUP as a factor are subsequently broken-down. To this end, we performed subsequent mixed ANOVA’s comparing the groups separately to identify which group drives the effect. As such, all comparisons between only two groups start from a significant overall interaction.*

*To clarify, we have now in subscript added the number of groups that was compared in the specific mixed ANOVA. Thus, GROUP_(3)_ in case all 3 groups were compared (overall interaction) and GROUP_(2)_ in case only 2 groups were compared (breaking-down an overall interaction).*

*We have now added the following sentences to the method section:*

*“When the overall mixed ANOVA revealed a significant interaction with GROUP as a factor, we performed subsequent mixed ANOVA’s to compare the GROUPS separately breaking-down this interaction. In these cases, the factor GROUP comprises only 2 levels. For the overall interaction we use “GROUP_(3)_“ (referring to 3 within-subject levels) and when breaking-down the overall interaction comparing 2 groups, we use “GROUP_(2)_“ (referring to 2 within-subject levels).”*

*Moreover, in the result section we have replaced GROUP with GROUP_(3)_ or GROUP_(2)_ following the description above.*

Could the type of switch (word to arrow vs arrow to word) be influenced in a different manner? Did the authors look at this?

*AU: It is indeed possible that the degree of reward motivational enhancement of task-switching differs per switch type. Effects of reward motivation are more readily observed on the most established task-set (i.e the arrow task) (Aarts et al., 2011). Therefore, we now performed extra analysis including TASK (i.e. arrow or word) as additional within-subject factor. In patients, in terms of error rates, there was no significant DRUG x REWARD x TRIAL-TYPE x TASK x GROUP interaction (F_(1,43)_=1.88, p=0.18, ηp^2^=0.042) and no significant DRUG x REWARD x TRIAL-TYPE x TASK interaction (F_(1,43)_=0.11, p=0.74, ηp^2^=0.003). Therefore, to compare patient data with healthy control data, we averaged patient data across drug sessions. This analysis revealed no differences between depressed patients, nondepressed patients and healthy controls in terms of the effect of task (i.e. arrow or word) on reward motivated cognitive control, indicated by a non-significant interaction between REWARD, TRIAL-TYPE, TASK and GROUP (F_(2,65)_=0.01, p=0.99, ηp^2^=0.000).*

*We performed a similar analysis for reaction times. Again in patients, there was no significant DRUG x REWARD x TRIAL-TYPE x TASK x GROUP interaction (F_(1,43)_=0.73, p=0.40, ηp^2^=0.017) and no significant DRUG x REWARD x TRIAL-TYPE x TASK interaction (F_(1,43)_=1.47, p=0.23, ηp^2^=0.033). Therefore, to compare patient data with healthy control data, we averaged patient data across drug sessions. This analysis revealed no differences between depressed patients, nondepressed patients and healthy controls in terms of the effect of task (i.e. arrow or word) on reward motivated cognitive control, indicated by a non-significant interaction between REWARD, TRIAL-TYPE, TASK and GROUP (F_(2,65)_=0.00, p=0.99, ηp^2^=0.000).*

*With respect to error rates, we have now added the following sentences to the result section:*

*“Previously, we have shown that effects of reward motivation are more readily observed on the most established task-set (i.e. the arrow task) (Aarts et al., 2011). Therefore, we performed an extra analysis including TASK (i.e. arrow or word) as additional within-subject factor. In patients, in terms of error rates, there was no significant DRUG x REWARD x TRIAL-TYPE x TASK x GROUP interaction (F_(1,43)_=1.88, p=0.18, ηp^2^=0.042) and no significant DRUG x REWARD x TRIAL-TYPE x TASK interaction (F_(1,43)_=0.11, p=0.74, ηp^2^=0.003). Therefore, to compare patient data with healthy control data, we averaged patient data across drug sessions. Comparison of depressed patients, nondepressed patients and healthy controls revealed no differences in terms of the effect of task (i.e. arrow or word) on reward motivated cognitive control, indicated by a non-significant interaction between REWARD, TRIAL-TYPE, TASK and GROUP (F_(2,65)_=0.01, p=0.99, ηp^2^=0.000).”*

*With respect to reaction times, we have now added the following sentences to the result section:*

*“Again, we performed an extra analysis including TASK (i.e. arrow or word) as additional within-subject factor. In patients, in terms of reaction times, there was no significant DRUG x REWARD x TRIAL-TYPE x TASK x GROUP interaction (F_(1,43)_=0.73, p=0.40, ηp^2^=0.017) and no significant DRUG x REWARD x TRIAL-TYPE x TASK interaction (F_(1,43)_=1.47, p=0.23, ηp^2^=0.033). Therefore, to compare patient data with healthy control data, we averaged patient data across drug sessions. Comparison of depressed patients, nondepressed patients and healthy controls revealed no differences in terms of the effect of task (i.e. arrow or word) on reward motivated cognitive control, indicated by a non-significant interaction between REWARD, TRIAL-TYPE, TASK and GROUP (F_(2,65)_=0.00, p=0.99, ηp^2^=0.000).”*

**References**

Aarts, E., Helmich, R.C., Janssen, M.J., Oyen, W.J., Bloem, B.R. & Cools, R. (2011) Aberrant reward processing in Parkinson's disease is associated with dopamine cell loss. *Neuroimage*.

Olino, T.M., McMakin, D.L., Morgan, J.K., Silk, J.S., Birmaher, B., Axelson, D.A., Williamson, D.E., Dahl, R.E., Ryan, N.D. & Forbes, E.E. (2014) Reduced reward anticipation in youth at high-risk for unipolar depression: A preliminary study. *Developmental Cognitive Neuroscience*, **8**, 55-64.

Yang, X.H., Huang, J., Zhu, C.Y., Wang, Y.F., Cheung, E.F.C., Chan, R.C.K. & Xie, G.R. (2014) Motivational deficits in effort-based decision making in individuals with subsyndromal depression, first-episode and remitted depression patients. *Psychiatry Res.*, **220**, 874-882.

2^nd^ Editorial Decision	27/07/18

_____________________________________________________________________________________

Enhanced motivation of cognitive control in Parkinson’s disease

Dear Dr. Timmer,

Your resubmitted manuscript has been assessed by the Section Editor,  Dr. Yoland Smith, and ourselves.  We are please to say that it will be accepted for publication in EJN after you have dealt with a few minor points.

- Please supply a Graphical Abstract.
- Please proof-read the reference list, there are lots of errors and inconsistencies.  e.g. Kehagia et al; extraneous text associated with journal name for Rosier et al; sentence case should be used for titles but for some you have title case and some have all capitals.

Once done, we will send it off to production.

When finalized, please upload your complete revised manuscript onto the website, as a Word file (.doc, or .docx).  Please also ensure that a complete set of tables and figures is included as separate files, even if these have not changed from the originals.  At this stage it is necessary to provide high resolution figures.  Please see important instructions below.

Please go into https://mc.manuscriptcentral.com/ejn - Author Centre - manuscripts with decisions where you will find a 'create a revision' link under 'actions'. We ask that you please indicate the way in which you have responded to the points raised by the Editors and Reviewers in a letter. Please upload this response letter as a separate Word (.doc or PDF) file using the file designation "Authors' Response to Reviewers" when uploading your manuscript files.  Please DO NOT submit your revised manuscript as a new one.  Also, please note that only the  Author who submitted the original version of the manuscript should submit a revised version.

If you are able to respond fully to the points raised, we would be pleased to receive a revision of your paper within 30 days.

Thank you for submitting your work to EJN.

Best wishes,

Paul Bolam & John Foxe
co-Editors in Chief, EJN

_____________________________________________________________________________________

Authors’ Response	17/08/2018

_____________________________________________________________________________________

**Response letter**

Ref.: EJN-2018-01-25237

Enhanced motivation for cognitive control in Parkinson’s disease

European Journal of Neuroscience

Dear editor,

We are very pleased to hear that our manuscript will be accepted for publication after having dealt with a few minor point. We have now provided a graphical abstract and graphical abstract figure. Moreover, we have proof-read the reference list and dealt with the errors and inconsistencies.

Best wishes,

Monique Timmer, Esther Aarts, Rianne Esselink and Roshan Cools
